# Comparative long-term risks of chronic kidney disease and dialysis following conservative treatment, renal artery embolization, or nephrectomy in patients with blunt kidney injuries: retrospective cohort study

**DOI:** 10.1093/bjsopen/zrag051

**Published:** 2026-06-01

**Authors:** Jhih-Ming Tang, Ling-Wei Kuo, Jen-Fu Huang, Chih-Po Hsu, Chi-Tung Cheng, Huan-Wu Chen, Cheng-Hsien Wu, Yon-Cheong Wong, Szu-An Chen, Chia-Cheng Wang, Yu-San Tee, Chun-Hsiang Ou Yang, Chih-Yuan Fu, Chien-Hung Liao

**Affiliations:** Division of Trauma and Emergency Surgery, Jen-Ai Hospital, Dali Branch, Taichung, Taiwan; Division of Trauma and Emergency Surgery, Chang Gung Memorial Hospital, Linkou Medical Center, Taoyuan, Taiwan; School of Medicine, Chang Gung University, Taoyuan, Taiwan; Division of Trauma and Emergency Surgery, Jen-Ai Hospital, Dali Branch, Taichung, Taiwan; Division of Trauma and Emergency Surgery, Chang Gung Memorial Hospital, Linkou Medical Center, Taoyuan, Taiwan; School of Medicine, Chang Gung University, Taoyuan, Taiwan; Division of Trauma and Emergency Surgery, Chang Gung Memorial Hospital, Linkou Medical Center, Taoyuan, Taiwan; School of Medicine, Chang Gung University, Taoyuan, Taiwan; Division of Trauma and Emergency Surgery, Chang Gung Memorial Hospital, Linkou Medical Center, Taoyuan, Taiwan; School of Medicine, Chang Gung University, Taoyuan, Taiwan; School of Medicine, Chang Gung University, Taoyuan, Taiwan; Division of Emergency and Critical Care Radiology, Department of Medical Imaging and Intervention, Chang Gung Memorial Hospital, Linkou Medical Center, Taoyuan, Taiwan; School of Medicine, Chang Gung University, Taoyuan, Taiwan; Division of Emergency and Critical Care Radiology, Department of Medical Imaging and Intervention, Chang Gung Memorial Hospital, Linkou Medical Center, Taoyuan, Taiwan; School of Medicine, Chang Gung University, Taoyuan, Taiwan; Division of Emergency and Critical Care Radiology, Department of Medical Imaging and Intervention, Chang Gung Memorial Hospital, Linkou Medical Center, Taoyuan, Taiwan; Division of Trauma and Emergency Surgery, Chang Gung Memorial Hospital, Linkou Medical Center, Taoyuan, Taiwan; School of Medicine, Chang Gung University, Taoyuan, Taiwan; Division of Trauma and Emergency Surgery, Chang Gung Memorial Hospital, Linkou Medical Center, Taoyuan, Taiwan; School of Medicine, Chang Gung University, Taoyuan, Taiwan; Division of Trauma and Emergency Surgery, Chang Gung Memorial Hospital, Linkou Medical Center, Taoyuan, Taiwan; School of Medicine, Chang Gung University, Taoyuan, Taiwan; Division of Trauma and Emergency Surgery, Chang Gung Memorial Hospital, Linkou Medical Center, Taoyuan, Taiwan; School of Medicine, Chang Gung University, Taoyuan, Taiwan; Division of Trauma and Emergency Surgery, Chang Gung Memorial Hospital, Linkou Medical Center, Taoyuan, Taiwan; School of Medicine, Chang Gung University, Taoyuan, Taiwan; Division of Trauma and Emergency Surgery, Chang Gung Memorial Hospital, Linkou Medical Center, Taoyuan, Taiwan; School of Medicine, Chang Gung University, Taoyuan, Taiwan

**Keywords:** renal preservation, competing risk analysis, end-stage kidney disease, trauma surgery, inverse probability of treatment weighting, abdominal injuries

## Abstract

**Background:**

Long-term changes in renal function following blunt kidney injury (BKI) remain insufficiently studied, particularly in patients who undergo renal artery embolization (RAE). The aim of this study was to clarify the long-term risks of chronic kidney disease (CKD) and dialysis in patients with BKI after unilateral nephrectomy, RAE, or conservative treatment. The hypothesis was that both unilateral nephrectomy and RAE would increase these risks compared with conservative treatment, with RAE posing a lower risk.

**Methods:**

This retrospective cohort study used data from Taiwan’s National Health Insurance Research Database from 2001 to 2019, focusing on patients with BKIs. Outcomes included long-term CKD risks, lifelong dialysis, and all-cause mortality. Inverse probability of treatment weighting was used to minimize confounding.

**Results:**

During the study, 19 013 patients were diagnosed with BKIs; of these, 12 709 were analysed (unilateral nephrectomy, 274 (2.1%); RAE, 510 (4.0%); conservative treatment, 11 925 (93.8%)). The mean(standard deviation) age was 39.7(17.1) years, 69.4% were male, and the mean follow-up duration was 10.7(5.2) years. Compared with conservative treatment, patients undergoing unilateral nephrectomy (subdistribution hazard ratio (SHR) 1.97; 95% confidence interval (c.i.) 1.86 to 2.10) or RAE (SHR 1.17, 95% c.i. 1.09 to 1.26) had a higher risk of CKD. In addition, patients in the nephrectomy and RAE groups had an increased risk of lifelong dialysis, with an SHR of 3.97 (95% c.i. 3.09 to 5.10) and 1.46 (95% c.i. 1.07 to 1.99), respectively. Unilateral nephrectomy was associated with a higher risk of all-cause mortality than RAE and conservative treatment, with hazard ratios of 1.14 (95% c.i. 1.06 to 1.23) and 1.63 (95% c.i. 1.52 to 1.75), respectively.

**Conclusions:**

Patients with BKIs are at higher risk of long-term CKD and potential lifelong dialysis after both unilateral nephrectomy and RAE, with RAE presenting a lower risk than nephrectomy. Patients with BKIs who undergo unilateral nephrectomy or RAE require follow-up to monitor renal function and receive health education on kidney protection.

## Teaser

Using a large, population-based retrospective cohort and inverse probability of treatment weighting, this study compared long-term renal outcomes (chronic kidney disease and dialysis) after conservative treatment, renal artery embolization (RAE), or nephrectomy for blunt kidney injuries. Nephrectomy carried a significantly higher risk of chronic kidney disease and dialysis than both RAE and conservative management, whereas RAE was associated with a greater risk of both than conservative management.

## Introduction

Blunt kidney injuries (BKIs) occur in 1–5% of trauma patients, a relatively low incidence attributed to the kidney’s well-protected retroperitoneal position^1–5^. BKI typically results from high-energy incidents, such as motor vehicle accidents, falls from heights, or direct blows to the flank, where rapid deceleration or direct compression leads to renal parenchymal laceration, subcapsular haematoma, or injury to the renal pedicle^6,7^. Clinically, BKI presents with a spectrum of signs including flank pain, ecchymosis, and haematuria^7,8^. Diagnosis relies on clinical suspicion from the mechanism of injury, the presence of either gross or microscopic haematuria, focused assessment with sonography for trauma, or computed tomography in patients with stable haemodynamics^5,9,10^. Computed tomography is the imaging modality of choice because of its ability to identify injury grade and active bleeding^11–13^. The American Association for the Surgery of Trauma grading system provides an essential anatomic framework for management, where low-grade injuries (grades I–II) are typically managed conservatively, whereas high-grade injuries (grades III–V) involving deep lacerations, urinary extravasation, or vascular compromise often necessitate interventional radiology or surgical procedures^13,14^.

Haemodynamic stability is the primary concern in treatment. Non-operative management, including close monitoring, serial blood tests, and renal artery embolization (RAE) as an adjunct to achieve haemostasis, is currently the mainstay of treatment for stable patients^13,14^. Non-operative management can be attempted in 83–90% of patients with BKIs, with a reported success rate of 94–97%^5,15,16^. Conversely, for patients who do not respond to resuscitation, prompt surgical exploration is essential^13,14^. In these critical situations, a nephrectomy may be required^17–19^. Despite the continued need for surgery in unstable patients, the management of BKI has shifted towards more conservative approaches. This is evidenced by clinical data from Japan and the USA, where nephrectomy rates for BKIs have decreased significantly, coinciding with a marked increase in the use of RAE as a minimally invasive alternative for haemorrhage control^20,21^.

High-grade renal trauma could lead to a loss of functional reserve in the kidney^22–24^. Furthermore, patients with BKIs following nephrectomy were found to have a higher long-term risk of CKD and lifelong dialysis^25^. However, with the growing use of RAE for patients with BKIs, the long-term risks of CKD and the need for lifelong dialysis remain uncertain. A study using the American College of Surgeons Trauma Quality Improvement Program databank revealed that both nephrectomy and RAE were associated with acute kidney injury during the index admission^26^. Additional evidence concerning the long-term risks following RAE is derived from studies employing serum creatinine measurements and various imaging modalities to evaluate the viability and morphological changes of embolized kidneys over time^27–30^. Notably, in the absence of a control group, it is challenging to evaluate the long-term risks associated with RAE. Other studies have had a relatively short follow-up period, making it difficult to clearly identify the associated risks^31,32^.

The aim of the present study was to clarify the long-term risks of CKD and dialysis in patients with BKIs after nephrectomy, RAE, or conservative treatment. The hypothesis was that both nephrectomy and RAE would increase these risks compared with conservative treatment, with RAE posing a lower risk.

## Methods

### Data source

Taiwan’s National Health Insurance system has been operational for three decades, providing mandatory coverage to over 99% of citizens. The programme supports a broad spectrum of medical costs for various illnesses, with the Taiwan Government acting as the sole payer. A peer review mechanism is in place to validate medical procedures. Moreover, the National Health Insurance includes a catastrophic illness programme that addresses severe health issues, such as major trauma with an injury severity score (ISS) ≥ 16 and end-stage renal disease requiring chronic dialysis. Patients eligible for this programme receive comprehensive coverage, with substantially reduced out-of-pocket expenses. This study used the National Health Insurance Research Database (NHIRD), which gathers claims data for insurance reimbursements. The NHIRD, along with its associated databases, is administered by the Health and Welfare Data Science Centre as a component of the National Health Informatics Project. Additional details about the NHIRD and its validation processes are available elsewhere^33–35^. This study was approved by the Institutional Review Board of the Chang Gung Medical Foundation (IRB No. 202101615B0), and the requirement for informed consent was waived due to its retrospective nature.

### Study design

The data used in the present study spanned from 2001 to 2019. Participants were identified in the NHIRD using specific diagnostic codes for BKI. The International Classification of Diseases (ICD), Ninth Revision, Clinical Modification codes (866.0X; excluding penetrating injury code 866.1X) were used for patients before 31 December 2015, and transitioned to ICD, Tenth Revision, Clinical Modification codes (S37.0X; excluding penetrating injury code S31.6X) thereafter. A comprehensive list of all ICD codes used for participant selection and the identification of co-morbidities is provided in *Table S1*. Patients with missing demographic data, such as sex and age, and individuals aged < 18 years were excluded from the study. Patients with a history of CKD or those who were receiving chronic dialysis or had undergone nephrectomy, partial nephrectomy, renal transplantation, or renal donation before the trauma or within 1 year of discharge were also excluded. Furthermore, individuals who underwent bilateral nephrectomy or partial nephrectomy during the index admission were ineligible for inclusion. Bilateral nephrectomy was excluded because it necessitates immediate, permanent dialysis, which represents a clinical course distinct from the gradual CKD progression under investigation. Partial nephrectomy was also excluded to minimize potential bias; the NHIRD lacks granular data to quantify the extent of resected parenchyma or the impact of operative factors, such as warm ischaemia time, which significantly affect long-term renal outcomes^36,37^. Patients with concurrent moderate to severe liver or spleen injuries, as well as unstable pelvic fractures, were excluded owing to inadequate information regarding the site of transarterial embolization^38^. The exclusion criteria also included trauma-related deaths during the initial hospitalization and patients with < 365 days of follow-up after discharge, including those who died within this period. Eligible patients were classified into three groups based on treatment modality: unilateral nephrectomy, RAE, or conservative treatment.

### Measurement of covariates

Basic demographic data, including patient age and sex, were recorded. Injuries associated with the index BKI admission, including traumatic brain injury, spinal cord injury, cardiac and pulmonary injuries, pneumothorax, haemothorax, gastrointestinal injuries, femur fractures, and major trauma with an ISS > 16, were documented. In addition, any co-morbidities (such as hypertension, ischaemic heart disease, congestive heart failure, ischaemic stroke, diabetes, cirrhosis, and chronic obstructive pulmonary disease) were noted. Co-morbidities were considered to be present if there were at least two outpatient diagnoses or a single inpatient diagnosis within the year leading up to the index BKI admission. Bed confinement or paralysis may be associated with a higher risk of mortality^39^, whereas hypertension is strongly linked to the deterioration of renal function^40,41^. Therefore, whether patients were confined to bed or were paralysed and had a new diagnosis of hypertension during the first year after discharge was also assessed. The diagnostic accuracy of the ICD codes for the majority of the aforementioned co-morbidities has been previously validated in several studies using the NHIRD, ensuring the reliability of the clinical data^35,42^.

### Outcome definitions

The primary outcomes were the incidence and cumulative risks of newly diagnosed CKD, requirement for lifelong dialysis, and all-cause mortality. To reduce the risk of reverse causality, outcomes were assessed only when they occurred more than 1 year after the index admission^43^. Details on the causes, dates, and locations of death are available through the Taiwan Death Registry, maintained by the Health and Welfare Data Science Centre. New-onset CKD required two outpatient diagnoses spaced at least 90 days apart^44^. Chronic dialysis status was verified via the Catastrophic Illness Certificate database, where patients with a Catastrophic Illness Certificate card are exempt from all chronic dialysis costs^45^. Relevant ICD codes for these diseases are provided in *Table S1*.

### Statistical analysis

To mitigate potential confounding and ensure balanced baseline characteristics among the three study groups (that is, nephrectomy, RAE, and conservative treatment), an inverse probability of treatment weighting (IPTW) approach using propensity scores was applied. The propensity scores were calculated using generalized boosted modelling with 10 000 decision trees^46^. All covariates were used to compute the propensity scores, as detailed in *Table 1*. Importantly, the admission date for BKI was factored into the calculation of propensity scores to guarantee comparable potential follow-up durations among the three study groups. The balance of baseline characteristics among the three study groups was assessed before and after applying IPTW, using the maximum absolute standardized difference (MASD) as a measure. Although an MASD < 0.1 is often considered indicative of a negligible imbalance, a threshold of < 0.2 was adopted to represent a non-significant difference for studies involving multiple treatment groups, a criterion supported by established literature for propensity score-based analyses^46^ . As a result, all outcome comparisons between the study groups were performed on the IPTW-adjusted cohort. A Cox proportional hazards model was used to evaluate the risk of all-cause mortality among the three groups, whereas the Fine–Gray subdistribution hazard model was used to analyse kidney outcomes, treating all-cause mortality as a competing risk.

**Table 1:** Baseline demographics and characteristics of patients between different treatments for blunt kidney injuries before inverse probability of treatment weighting

	Total (*n* = 12 709)	Nephrectomy (*n* = 274)	RAE (*n* = 510)	Conservative (*n* = 11 925)	MASD
**Demographics**					
Age (years), mean(s.d.)	39.7(17.1)	38.5(17.7)	38.9(17.4)	39.8(17.0)	0.07
Age grouping					
< 30 years	4778 (37.6%)	117 (42.7%)	220 (43.1%)	4441 (37.2%)	0.12
30–49 years	4399 (34.6%)	86 (31.4%)	148 (29.0%)	4165 (34.9%)	0.13
50–64 years	2345 (18.5%)	42 (15.3%)	97 (19.0%)	2206 (18.5%)	0.10
≥ 65 years	1187 (9.3%)	29 (10.6%)	45 (8.8%)	1113 (9.3%)	0.06
Sex					0.11
Male	8825 (69.4%)	203 (74.1%)	364 (71.4%)	8258 (69.2%)	
Female	3884 (30.6%)	71 (25.9%)	146 (28.6%)	3667 (30.8%)	
**Associated injuries**					
Traumatic brain injury	473 (3.7%)	14 (5.1%)	43 (8.4%)	416 (3.5%)	0.21
Spinal cord injury	120 (0.9%)	0 (0.0%)	5 (1.0%)	115 (1.0%)	0.14
Injury to heart and lung	470 (3.7%)	13 (4.7%)	43 (8.4%)	414 (3.5%)	0.21
PTX and HTX	1334 (10.5%)	55 (20.1%)	120 (23.5%)	1159 (9.7%)	0.38
GI tract injury	361 (2.8%)	41 (15.0%)	19 (3.7%)	301 (2.5%)	0.45
Femur fracture	281 (2.2%)	11 (4.0%)	19 (3.7%)	251 (2.1%)	0.11
Major trauma*	912 (7.2%)	105 (38.3%)	197 (38.6%)	610 (5.1%)	0.89
**Co-morbidities**					
Hypertension	1634 (12.9%)	34 (12.4%)	67 (13.1%)	1533 (12.9%)	0.02
Ischaemic heart disease	371 (2.9%)	8 (2.9%)	13 (2.6%)	350 (2.9%)	0.02
Congestive heart failure	86 (0.7%)	3 (1.1%)	3 (0.6%)	80 (0.7%)	0.06
Ischaemic stroke	251 (2.0%)	1 (0.4%)	9 (1.8%)	241 (2.0%)	0.15
Diabetes	192 (1.5%)	4 (1.5%)	12 (2.4%)	176 (1.5%)	0.07
Cirrhosis	108 (0.9%)	0 (0.0%)	8 (1.6%)	100 (0.8%)	0.18
COPD	250 (2.0%)	5 (1.8%)	5 (1.0%)	240 (2.0%)	0.08
Confined to bed	100 (0.8%)	6 (2.2%)	8 (1.6%)	86 (0.7%)	0.12
Hypertension diagnosed during the first year of follow-up	1776 (14.0%)	31 (11.3%)	89 (17.5%)	1656 (13.9%)	0.18

Values are *n* (%) unless otherwise stated. *Major trauma was defined as an injury severity score ≥ 16. RAE, renal artery embolization; MASD, maximum absolute standardized difference; s.d., standard deviation; PTX, pneumothorax; HTX, haemothorax; GI, gastrointestinal; COPD, chronic obstructive pulmonary disease.

Apart from missing demographic data (that is, age and sex), no additional missing values were identified for the covariates or outcomes. Two-tailed *P* < 0.050 was deemed statistically significant. All statistical analyses were conducted using R version 4.2.1 (R Foundation for Statistical Computing, Vienna, Austria) and SAS® version 9.4 (SAS Institute, Cary, NC, USA).

## Results

### Patient selection and temporal trends

**Fig. 1:**
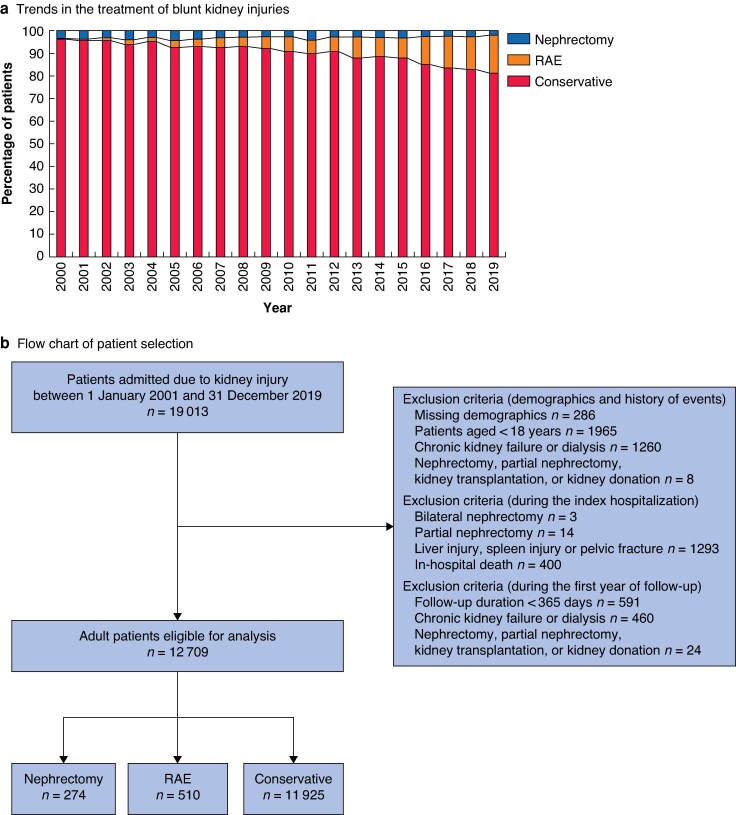
Temporal trends in the treatment of blunt kidney injuries throughout the study period and flow chart of patient selection **a** Trends in the treatment of blunt kidney injuries over the study period. **b** Flow chart of patient selection, detailing inclusion and exclusion criteria. RAE, renal artery embolization.

Over the study period, 19 013 patients were diagnosed with BKIs. Of these patients, 12 709 were considered eligible for analysis in the present study: 274 patients (2.1%) who underwent nephrectomy, 510 patients (4.0%) who underwent RAE, and 11 925 patients (93.8%) who were treated conservatively. RAE has emerged as the primary haemostatic procedure for BKIs over the years, as disclosed in *Fig. 1a*. The inclusion process for the study is shown in *Fig. 1b*.

### Baseline characteristics

As indicated in *Table 1*, the study group was relatively young, with a mean(standard deviation (s.d.)) age of 39.7(17.1) years, and was predominantly male (69.4%). The incidence of traumatic brain injury was higher in the RAE than nephrectomy and conservative treatment groups (8.4 *versus* 5.1 and 3.5%, respectively; MASD = 0.21), as was the incidence of haemopneumothorax (23.5 *versus* 20.1 and 9.7%, respectively; MASD = 0.38). Conversely, patients in the nephrectomy group were more likely to have gastrointestinal tract injuries than those in the RAE or conservative treatment groups (15.0 *versus* 3.7 and 2.5%, respectively; MASD = 0.45). The rate of major trauma, defined as an ISS ≥ 16, in the nephrectomy, RAE, and conservative treatment groups was 38.3%, 38.6%, and 5.1%, respectively (MASD = 0.89). The prevalence of co-morbidities was similar among the three study groups. The overall mean(s.d.) follow-up duration was 10.7(5.2) years. Before IPTW, the mean(s.d.) follow-up duration in the nephrectomy, RAE, and conservative treatment groups was 10.3(5.1), 7.1(4.3), and 10.8(5.2) years, respectively. After applying IPTW, all baseline characteristics, including the follow-up duration, in three groups were comparable, with all MASD values below 0.20 (*Table S2*).

### Risk of CKD

**Fig. 2:**
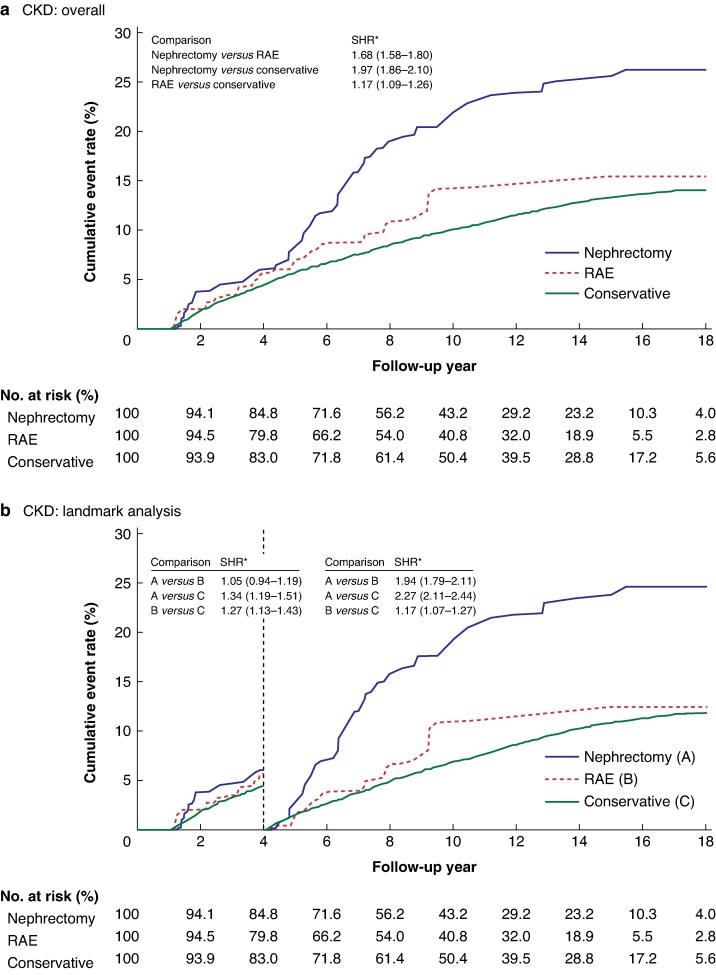
Cumulative event rates of CKD among different treatment groups after inverse probability of treatment weighting Cumulative CKD event rates **a** overall during the entire follow-up period and **b** in the landmark analysis (at 4 years of follow-up). *Values in parentheses are 95% confidence intervals. CKD, chronic kidney disease; RAE, renal artery embolization; SHR, subdistribution hazard ratio.

*Table 2* presents long-term follow-up outcomes related to CKD for the three study groups. The risk of CKD was higher in both the nephrectomy (subdistribution hazard ratio (SHR) 1.97; 95% confidence interval (c.i.) 1.86 to 2.10) and RAE (SHR 1.17, 95% c.i. 1.09 to 1.26) groups than in the conservative treatment group. Patients who underwent nephrectomy also had a higher risk of CKD than those who underwent RAE (SHR 1.68; 95% c.i. 1.58 to 1.80). *Figure 2a* illustrates the long-term risk of CKD across the three groups. Because group differences began to diverge around the fourth to fifth years of follow-up, a landmark analysis was conducted in the fourth year of follow-up (*Fig. 2b*). This landmark analysis revealed no significant difference in CKD risk between the nephrectomy and RAE groups during the first 4 years of follow-up. However, the observed difference in CKD risk between the groups persisted beyond the fourth year of follow-up.

**Table 2:** Follow-up outcomes of patients between different treatments for blunt kidney injuries in the IPTW-adjusted cohort

	Event rate (%)	Incidence rate (no. of events/10 000 person-years)	HR/SHR*	
			RAE	Nephrectomy
**Chronic kidney disease**				
Conservative	14.1	141.0 (134.5, 147.5)	1.17 (1.09, 1.26)†	1.97 (1.86, 2.10)†
RAE	15.4	174.2 (165.0, 183.3)		1.68 (1.58, 1.80)†
Nephrectomy	26.2	283.0 (271.6, 294.3)		
**Chronic dialysis**				
Conservative	0.6	6.0 (4.7, 7.3)	1.46 (1.07, 1.99)†	3.97 (3.09, 5.10)†
RAE	0.9	8.8 (6.9, 10.8)		2.71 (2.10, 3.50)†
Nephrectomy	2.6	24.6 (21.5, 27.7)		
**All-cause death**				
Conservative	10.7	100.5 (95.1, 105.8)	1.43 (1.33, 1.55)†	1.63 (1.52, 1.75)†
RAE	13.7	140.9 (133.0, 148.7)		1.14 (1.06, 1.23)†
Nephrectomy	17.7	163.0 (155.1, 170.9)		

Values in parentheses are 95% confidence intervals. After IPTW, the number of weighted patients in the conservative, nephrectomy, and RAE groups was 12 670.8, 9030.3, and 9149.6, respectively. *Column *versus* row. IPTW, inverse probability of treatment weighting; HR, hazard ratio; SHR, subdistribution hazard ratio; RAE, renal artery embolization. †*P* < 0.050.

### Lifelong dialysis

**Fig. 3:**
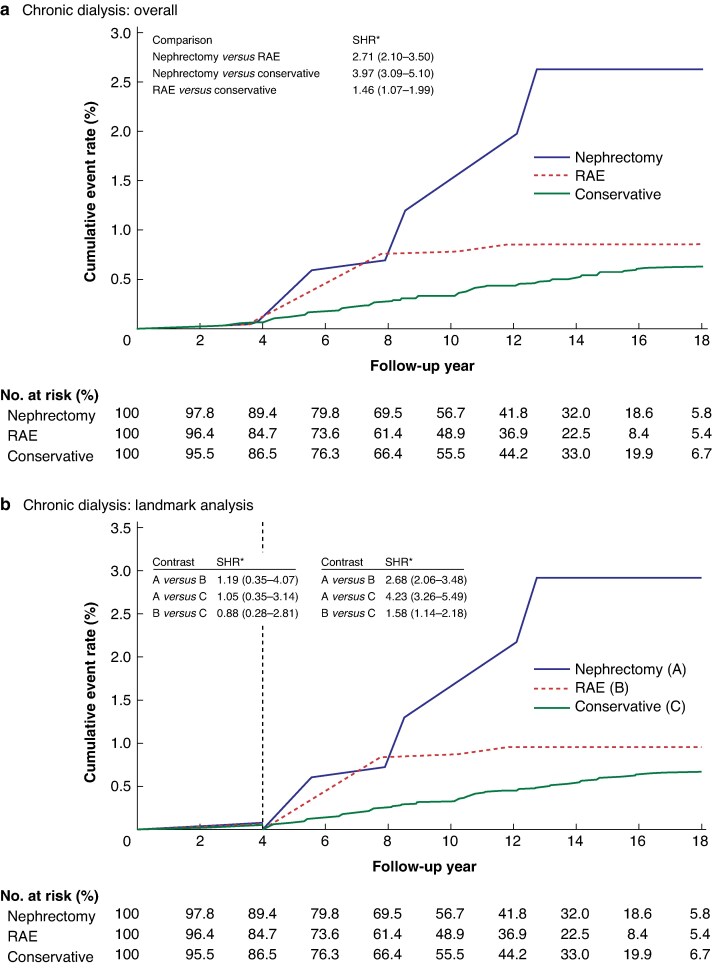
Cumulative event rates of the need for lifelong dialysis among different treatment groups after inverse probability of treatment weighting Chronic dialysis event rates **a** overall during the entire follow-up period and **b** in the landmark analysis (at 4 years of follow-up). *Values in parentheses are 95% confidence intervals. RAE, renal artery embolization; SHR, subdistribution hazard ratio.

Patients with BKIs who underwent nephrectomy (SHR 3.97; 95% c.i. 3.09 to 5.10) and those in the RAE group (SHR 1.46; 95% c.i. 1.07 to 1.99) had a higher risk of lifelong dialysis than those who underwent conservative treatment. The risk of lifelong dialysis was higher in the nephrectomy than RAE group (SHR 2.71; 95% c.i. 2.10 to 3.50), as shown in *Fig. 3a*. A 4-year latency in the development of lifelong dialysis was also noted (*Fig. 3b*). In the first 4 years after the index admission, the risk of lifelong dialysis was similar among the three study groups. However, the risk of lifelong dialysis started to diverge among the treatment groups in the fourth year of follow-up. Notably, patients who underwent unilateral nephrectomy had a four-fold increase in the risk of lifelong dialysis after this timepoint (SHR 4.23; 95% c.i. 3.26 to 5.49) compared with the conservative group.

### All-cause mortality

**Fig. 4:**
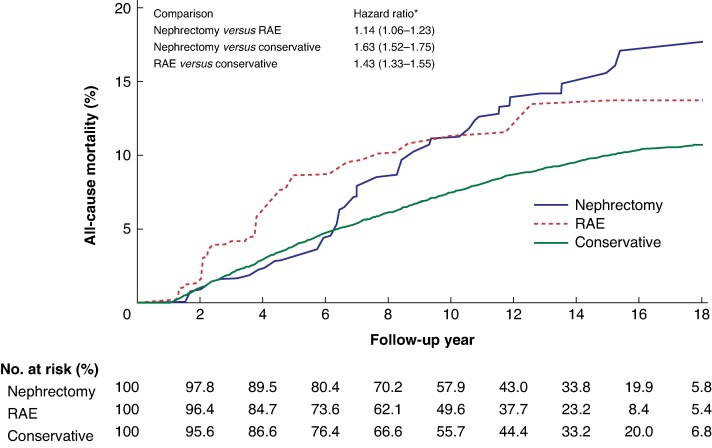
Cumulative event rates of all-cause mortality among different treatment groups after inverse probability of treatment weighting *Values in parentheses are 95% confidence intervals. RAE, renal artery embolization.

The risk of all-cause mortality was higher in patients who underwent nephrectomy than in those who underwent RAE (hazard ratio (HR) 1.14; 95% c.i. 1.06 to 1.23) or conservative treatment (HR 1.63; 95% c.i. 1.52 to 1.75). The mortality risk was higher in the RAE group than in the conservative treatment group (HR 1.43; 95% c.i. 1.33 to 1.55; *Fig. 4*). The cumulative mortality and incidence rates are detailed in *Table 2*.

## Discussion

The present study revealed that both unilateral nephrectomy and RAE significantly increased the long-term risk of CKD and the need for lifelong dialysis; however, RAE had a comparatively smaller impact on adverse renal outcomes. In addition, patients who underwent nephrectomy had a higher risk of all-cause mortality. RAE preserves renal function effectively and should be considered the preferred method for patients with BKIs experiencing active bleeding when clinically appropriate.

RAE has assumed an increasingly prominent role in the management of BKIs. In Japan, the use of RAE increased markedly from 25.9% in 2004–2006 to 35.5% in 2016–2018, whereas the nephrectomy rate declined from 5.3 to 2.4% over the same period^21^. Similarly, in the USA, the proportion of RAEs increased markedly from 1.4% in 2002 to 53.3% in 2012, with the nephrectomy rate decreasing from 8.2 to 2.1% over the same time frame^45–51^. Advances in resuscitation and critical care have enabled clinicians to stabilize patients more effectively, thereby reducing the need for nephrectomies. Furthermore, the increased availability of interventional radiologists, along with the maturation and improved accessibility of techniques, has contributed significantly to these trends. However, the rise in RAE use far outpaces the decline in nephrectomy rates, suggesting that some patients who could have been successfully managed with conservative treatment may have undergone unnecessary RAE. According to the present study, conservative treatment remains the approach associated with the fewest long-term side effects, and clinicians should base their treatment decisions on appropriate indications rather than indiscriminate intervention.

The association between unilateral nephrectomy and the long-term risk of CKD or the need for lifelong dialysis remains controversial. Although numerous studies^47–53^ reported a non-significant relationship between unilateral nephrectomy and the long-term development of CKD and subsequent lifelong dialysis, these studies did observe lower creatinine clearance rates and increased proteinuria over time. Nevertheless, most of these studies had limited sample sizes to detect risks or did not compare the risks following unilateral nephrectomy to a control group. Cozzi *et al*.^54^ conducted a review of 22 papers that suggested unilateral nephrectomy during childhood increases the risk of lower renal function, consistent with the findings of the present study. Notably, most studies focused on oncological or donor nephrectomy, and research specifically addressing patients with BKI is scarce. The present study used a nationwide population-based data set to provide real-world data to gain a better understanding of this issue.

A single-vessel system supplies the kidneys; therefore, RAE can lead to parenchymal ischaemia and a loss of functional reserve. Imaging studies conducted 6–12 months after the procedure to evaluate parenchymal ischaemia revealed a mean parenchymal perfusion deficit of 5–6%^29^. In another study, after a mean follow-up of 4.6 years, radioisotope renography indicated functional improvement in the embolized renal units^31^. Despite the evidence from imaging studies, there has been no long-term follow-up or assessment of the risks associated with RAE for BKIs. Only one study reported a mean follow-up of 2.7 years, finding no changes in renal function among patients with grade 5 kidney injuries following RAE, based on questionnaire responses^31^. The present study provides insights into the long-term prognosis for patients with BKIs who underwent RAE.

BKIs can lead to a loss of renal function, particularly in cases of high-grade or vascular injury^53^. There is also a loss of functional renal reserve, which is necessary for haemostasis and life-saving interventions, after RAE and nephrectomy. Brenner^55^ proposed that the permanent loss of nephron units leads to structural and functional adaptations in the surviving nephrons, which initially compensate for this loss through hypertrophy and elevated intraglomerular pressures. However, hyperfiltration in the remaining nephrons can damage the glomeruli and contribute to the deterioration of renal function^56^. This may explain the findings of the present study, namely that both nephrectomy and RAE were associated with long-term adverse renal outcomes after a latency period of approximately 4 years.

Compared with patients with BKIs who underwent conservative treatment, those who underwent unilateral nephrectomy had a higher long-term risk of all-cause mortality, whereas the RAE group had a comparatively lower risk. A detailed analysis of the causes of death was not conducted in the present study. Several studies^57–59^ have reported that unilateral nephrectomy and the resulting chronic renal insufficiency are associated with an increased risk of cardiovascular disease and mortality, which supports the findings of the present study.

Life-saving measures remain the top priority in managing BKIs. Clinicians should adhere to current management guidelines to maximize survival and long-term renal outcomes. With RAE and nephrectomy performed for haemostasis and life-saving purposes, patients must be informed about the long-term risks of CKD and the possibility of requiring lifelong dialysis. Health education on kidney protection should include avoiding nephrotoxic agents, quitting smoking, and controlling blood pressure and blood sugar levels^60,61^. Clinicians should also be aware that patients with BKIs who have undergone unilateral nephrectomy have an increased risk of all-cause mortality.

This study has some limitations. First, as a retrospective cohort study, the results should be interpreted as showing associations rather than establishing causality. Second, the nature of a database study means that certain detailed information may be lacking. For example, the NHIRD lacks granular clinical information, such as the American Association for the Surgery of Trauma renal injury grade and acute physiological data including vital signs (for example, blood pressure and heart rate) and body mass index (obesity). Although major trauma (ISS ≥ 16) and multiple chronic co-morbidities were adjusted for to minimize bias, the potential for residual confounding related to the specific severity of the renal injury or baseline physiological reserve cannot be entirely ruled out. Moreover, information on the exact location of transarterial embolization was not available, and the approach of identifying patients undergoing RAE by excluding other sites could have introduced bias. Furthermore, the absence of patients’ laboratory data prevented assessment of the severity of renal dysfunction. Third, the exclusion process in this study may have introduced a risk of bias into the findings. In addition, because the findings are based on the Taiwanese population and treatment practices, they may not be directly generalizable to other populations. A future prospective study incorporating long-term clinical follow-up, laboratory tests, and imaging results may be necessary to draw more definitive conclusions.

For patients with BKIs, both unilateral nephrectomy and RAE carry a higher risk of long-term CKD than conservative management alone, as well the potential need for lifelong dialysis; however, the risks are lower for RAE than unilateral nephrectomy. Clinicians should aim to enhance survival and long-term renal outcomes when managing individuals with BKI in accordance with current practice guidelines.

## Supplementary Material

zrag051_Supplementary_Data

## Data Availability

The data that support the findings of this study were obtained from the National Health Insurance Research Database in Taiwan. Restrictions apply to the availability of these data, which were used under licence for the present study and are not publicly available.
