# Supplementary material for: Comparative long-term risks of chronic kidney disease and dialysis following conservative treatment, renal artery embolization, or nephrectomy in patients with blunt kidney injuries: retrospective cohort study
Source: BJS Open. 2026 Jun 1;10(3):zrag051. doi: 10.1093/bjsopen/zrag051 (PMC13224839; doi:10.1093/bjsopen/zrag051)
Supplement: zrag051_Supplementary_Data [file zrag051_supplementary_data.docx]

**Comparative Long-term Risks of Chronic Kidney Disease and Dialysis Following Conservative Treatment, Renal Artery Embolization, or Nephrectomy in Patients with Blunt Kidney Injuries: Retrospective Cohort Study**

Jhih-Ming Tang^a^; Ling-Wei Kuo^b, c^; Jen-Fu Huang^a, b, c^; Chih-Po Hsu^b, c^; Chi-Tung Cheng^b, c*^; Huan-Wu Chen^c,d^; Cheng-Hsien Wu^c,d^; Yon-Cheong Wong^c,d^; Szu-An Chen^b, c^; Chia-Cheng Wang^b, c^; Yu-San Tee^b, c^; Chun-Hsiang Ou Yang^b, c^; Chih-Yuan Fu^b, c^; Chien-Hung Liao^b, c^

^a^ Division of Trauma and Emergency Surgery, Jen-Ai Hospital, Dali Branch, Taichung, Taiwan

^b^ Division of Trauma and Emergency Surgery, Chang Gung Memorial Hospital, Linkou Medical Center, Taoyuan, Taiwan

^c^ Chang Gung University, Taoyuan, Taiwan

^d^ Division of Emergency and Critical Care Radiology, Department of Medical Imaging and Intervention, Chang Gung Memorial Hospital, Linkou Medical Center, Taoyuan, Taiwan

**Corresponding author**: Chi-Tung Cheng, MD, Ph.D.

Division of Trauma and Emergency Surgery, Chang Gung Memorial Hospital, Linkou Medical Center, No. 5, Fu-Hsing Street, Guishan District, Taoyuan City, 333, Taiwan, (R.O.C.)

Tel: +886-3-3281200 Ext. 3651; Fax: +886-3-3289582;

E-mail address: [atong89130@gmail.com](mailto:atong89130@gmail.com)

**Supplementary Materials - Index**

| **Supplementary Figures and Tables** |  |
| --- | --- |
| Supplementary Table S1 | *page 2* |
| Supplementary Table S2 | *page 3* |

**Supplementary Figures and Tables**

**Supplementary Table S1.** ICD diagnostic codes used in the study

| Disease | ICD-9 | ICD-10 |
| --- | --- | --- |
| Kidney injury | 866, not 866.1 | S37.0x, not S31.6 |
| Unstable pelvic fracture | 808.43, 808.53 | S32.811A, S32.810B |
| Spleen injury (moderate to severe) | 865.03, 865.04 | S36.021A, S36.031A, S36.032A, S36.021S, S36.031S, S36.032S, S36.021D, S36.031D, S36.032D |
| Liver injury (moderate to severe) | 864.03, 864.04 | S36.115A, S36.116A, S36.115S, S36.116S, S36.115D, S36.116D |
| Associated injuries |  |  |
| Traumatic brain injury | 852, 853 | S06.4, S06.5, S06.6 |
| Spinal cord injury | 806 | S14 |
| Injury to heart and lung | 861 | S26, S27.3 |
| Traumatic pneumothorax and hemothorax | 860 | S27.0, S27.1, S27.2 |
| Injury to gastrointestinal tract | 863 | S36.3, S36.4, S36.5, S36.6 |
| Femur fracture | 820, 821 | S72 |
| Major trauma (ISS over 16) | 959.99 | T07 |
| Comorbidity |  |  |
| Hypertension | 401-405 | I10-I15, N26.2x |
| Ischemic heart disease | 410-414 | I20-I24 |
| Diabetes mellitus | 250 | E08-E13 |
| Chronic obstructive pulmonary disease | 491, 492, 496 | J41-J44 |
| Cirrhosis | 571.2x, 571.5x, 571.6x | K70.30, K74.0, K74.60, K74.69, K74.3, K74.4, K74.5 |
| Bed confinement status or paralysis | 334.xx, 341.xx, 342.xx, 344.xx, V49.89 | Z74.01, G11, G36, G37, G81, G82, G83 |
| Chronic kidney disease (exclusion and outcome) | 580-589, 403-404, 016.0x, 095.4x, 236.9x, 250.4x, 274.1x, 442.1x, 447.3x, 440.1x, 572.4x, 642.1x, 646.2x, 753.1x, 283.11, 403.01, 404.02, 446.21 | A18.11x, D59.3x, E10.2x, E11.2x, E13.2x, I12, I13, K76.7x, M10.3x, M31.0x, N00-N08, N14, N15.0x, N15.8x, N15.9x, N16, N17.1x, N17.2x, N18, N19, N20.0x, N25, N26.1x, N26.9x, N27, Q61 |

Abbreviation: ICD, International Classification of Diseases.

**Supplementary Table S2.** Baseline demographics and characteristics of patients between different treatments for blunt kidney injuries before and after IPTW

|  | Before IPTW† | | | | |  | After IPTW‡ | | | |
| --- | --- | --- | --- | --- | --- | --- | --- | --- | --- | --- |
| Variable | Total  (*n* = 12,709) | Nephrectomy  (*n* = 274) | RAE  (*n* = 510) | Conservative  (*n* = 11,925) | MASD |  | Nephrectomy  (*n* = 9,149.6) | RAE  (*n* = 9,030.3) | Conservative  (*n* = 12,670.8) | MASD |
| Demographics |  |  |  |  |  |  |  |  |  |  |
| Age, year | 39.7 ± 17.1 | 38.5 ± 17.7 | 38.9 ± 17.4 | 39.8 ± 17.0 | 0.07 |  | 39.5 ± 16.3 | 39.7 ± 16.8 | 39.7 ± 17.0 | 0.02 |
| Age grouping |  |  |  |  |  |  |  |  |  |  |
| <30 | 4,778 (37.6) | 117 (42.7) | 220 (43.1) | 4,441 (37.2) | 0.12 |  | 37.9 | 37.1 | 37.5 | 0.02 |
| 30-49 | 4,399 (34.6) | 86 (31.4) | 148 (29.0) | 4,165 (34.9) | 0.13 |  | 36.9 | 35.9 | 34.7 | 0.05 |
| 50-64 | 2,345 (18.5) | 42 (15.3) | 97 (19.0) | 2,206 (18.5) | 0.10 |  | 16.7 | 17.6 | 18.5 | 0.05 |
| ≥65 | 1,187 (9.3) | 29 (10.6) | 45 (8.8) | 1,113 (9.3) | 0.06 |  | 8.6 | 9.3 | 9.4 | 0.03 |
| Male | 8,825 (69.4) | 203 (74.1) | 364 (71.4) | 8,258 (69.3) | 0.11 |  | 72.4 | 72.5 | 69.4 | 0.07 |
| Associated injuries |  |  |  |  |  |  |  |  |  |  |
| Traumatic brain injury | 473 (3.7) | 14 (5.1) | 43 (8.4) | 416 (3.5) | **0.21** |  | 5.8 | 5.4 | 3.7 | 0.10 |
| Spinal cord injury | 120 (0.9) | 0 (0.0) | 5 (1.0) | 115 (1.0) | 0.14 |  | 0.0 | 0.7 | 1.0 | 0.14 |
| Injury to heart and lung | 470 (3.7) | 13 (4.7) | 43 (8.4) | 414 (3.5) | **0.21** |  | 2.1 | 3.5 | 3.7 | 0.09 |
| PTX and HTX | 1,334 (10.5) | 55 (20.1) | 120 (23.5) | 1,159 (9.7) | **0.38** |  | 13.2 | 13.2 | 10.4 | 0.09 |
| GI tract injury | 361 (2.8) | 41 (15.0) | 19 (3.7) | 301 (2.5) | **0.45** |  | 3.4 | 4.0 | 2.8 | 0.07 |
| Femur fracture | 281 (2.2) | 11 (4.0) | 19 (3.7) | 251 (2.1) | 0.11 |  | 2.5 | 1.7 | 2.2 | 0.06 |
| Major trauma* | 912 (7.2) | 105 (38.3) | 197 (38.6) | 610 (5.1) | **0.89** |  | 7.6 | 8.5 | 6.9 | 0.06 |
| Comorbidities |  |  |  |  |  |  |  |  |  |  |
| Hypertension | 1,634 (12.9) | 34 (12.4) | 67 (13.1) | 1,533 (12.9) | 0.02 |  | 13.4 | 13.6 | 12.9 | 0.02 |
| Ischemic heart disease | 371 (2.9) | 8 (2.9) | 13 (2.6) | 350 (2.9) | 0.02 |  | 2.6 | 2.8 | 2.9 | 0.02 |
| Congestive heart failure | 86 (0.7) | 3 (1.1) | 3 (0.6) | 80 (0.7) | 0.06 |  | 0.7 | 0.5 | 0.7 | 0.03 |
| Ischemic stroke | 251 (2.0) | 1 (0.4) | 9 (1.8) | 241 (2.0) | 0.15 |  | 0.1 | 1.6 | 2.0 | 0.19 |
| Diabetes mellitus | 192 (1.5) | 4 (1.5) | 12 (2.4) | 176 (1.5) | 0.07 |  | 0.6 | 1.0 | 1.5 | 0.09 |
| Cirrhosis | 108 (0.9) | 0 (0.0) | 8 (1.6) | 100 (0.8) | 0.18 |  | 0.0 | 0.7 | 0.8 | 0.13 |
| COPD | 250 (2.0) | 5 (1.8) | 5 (1.0) | 240 (2.0) | 0.08 |  | 1.7 | 1.9 | 2.0 | 0.02 |
| Bed confinement status | 100 (0.8) | 6 (2.2) | 8 (1.6) | 86 (0.7) | 0.12 |  | 0.8 | 1.1 | 0.8 | 0.04 |
| Hypertension diagnosed during the first year of follow up | 1,776 (14.0) | 31 (11.3) | 89 (17.5) | 1,656 (13.9) | 0.18 |  | 11.4 | 14.2 | 13.9 | 0.08 |

Abbreviation: IPTW, inverse probability of treatment weighting; RAE, renal artery embolization; MASD maximum absolute standardized difference; PTX, pneumothorax; HTX, hemothorax; GI, gastrointestinal; COPD, chronic obstructive pulmonary disease;

† Data are presented as frequency (percentage) or mean ± standard deviation;

‡ Data are presented as percentage or mean ± standard deviation;

# Not included in the calculation of propensity scores;

* Injury severity score ≥16.
